# NOD/scid IL-2Rg^null ^mice: a preclinical model system to evaluate human dendritic cell-based vaccine strategies *in vivo*

**DOI:** 10.1186/1479-5876-10-30

**Published:** 2012-02-25

**Authors:** Stefani Spranger, Bernhard Frankenberger, Dolores J Schendel

**Affiliations:** 1 Institute of Molecular Immunology, German Research Center for Environmental Health, Helmholtz Zentrum München, Marchioninistrasse 25, 81377 Munich, Germany; 2 Clinical Cooperation Group "Immune Monitoring", German Research Center for Environmental Health, Helmholtz Zentrum München, Marchioninistrasse 25, 81377 Munich, Germany

**Keywords:** Antitumor immunity, CD8^+ ^T cells, Dendritic cells, Murine model system, TLR-activation, Vaccination

## Abstract

**Background:**

To date very few systems have been described for preclinical investigations of human cellular therapeutics *in vivo*. However, the ability to carry out comparisons of new cellular vaccines *in vivo *would be of substantial interest for design of clinical studies. Here we describe a humanized mouse model to assess the efficacy of various human dendritic cell (DC) preparations. Two reconstitution regimes of NOD/scid IL2Rg^null ^(NSG) mice with adult human peripheral blood mononuclear cells (PBMC) were evaluated for engraftment using 4-week and 9-week schedules. This led to selection of a simple and rapid protocol for engraftment and vaccine evaluation that encompassed 4 weeks.

**Methods:**

NSG recipients of human PBMC were engrafted over 14 days and then vaccinated twice with autologous DC via intravenous injection. Three DC vaccine formulations were compared that varied generation time *in vitro *(3 days versus 7 days) and signals for maturation (with or without Toll-like receptor (TLR)3 and TLR7/8 agonists) using MART-1 as a surrogate antigen, by electroporating mature DC with *in vitro *transcribed RNA encoding full length protein. After two weekly vaccinations, the splenocyte populations containing human lymphocytes were recovered 7 days later and assessed for MART-1-specific immune responses using MHC-multimer-binding assays and functional assessment of specific killing of melanoma tumor cell lines.

**Results:**

Human monocyte-derived DC generated *in vitro *in 3 days induced better MART-1-specific immune responses in the autologous donor T cells present in the humanized NSG mice. Moreover, consistent with our *in vitro *observations, vaccination using mature DC activated with TLR3 and TLR7/8 agonists resulted in enhanced immune responses *in vivo*. These findings led to a ranking of the DC vaccine effects *in vivo *that reflected the hierarchy previously found for these mature DC variations *in vitro*.

**Conclusions:**

This humanized mouse model system enables comparisons among different DC vaccine types to be rapidly assessed *in vivo*. In addition, *ex vivo *analyses of human CD3^+ ^T cells recovered from the spleens of these mice are also possible, including studies on lymphocyte subsets, Th1/Th2 polarization, presence of regulatory T cells and the impact of DC vaccination on their functions.

## Background

Dendritic cell (DC) vaccines hold high therapeutic potential for induction of antitumor immunity in cancer patients [1,2]. Many current cancer vaccines focus on mature DC (mDC) loaded with tumor-associated antigens (TAA) and injected intradermally to activate CD8^+ ^cytotoxic T lymphocytes (CTL). Monocyte-derived DC are commonly used in DC vaccine strategies.

Various methods have been developed for preparation of monocyte-derived mDC for clinical studies. These mostly rely on a 6-day protocol using IL-4 and GM-CSF to induce immature DC (iDC), followed by a 24 h maturation phase [3]. Published studies demonstrated that monocyte-derived DC generated over 2 or 3 days give comparable [4] or enhanced [5] immune responses *in vitro *compared to 7-day mDC. Many clinical trials have utilized a four-component cocktail (4C) containing IL-1β, IL-6, TNF-α and PGE_2 _for DC maturation [6]. The discovery that TLR agonists can optimally activate murine DC to secrete IL-12 [7] led to studies of the impact of TLR agonists on human mDC cytokine production [8-14]. Encouraging results have also been obtained in the first clinical trial using TLR-stimulated DC [15] as well as studies using TLR agonists as immune stimulatory adjuvants [16]. We also have described DC maturation cocktails using quinoline-like molecules, R848 or CL075, in 3-day and 7-day mDC [17,18]. *In vitro *studies of DC matured with TLR7/8 agonists, with or without poly (I:C) as a TLR3 agonist, resulted in substantial secretion of bioactive IL-12(p70) and high potential to activate innate and adaptive immune responses [17,18].

Humanized mouse models could provide a useful preclinical tool for assessment of immune responses *in vivo *[19] but reports characterizing immune responses after DC-based vaccination are rare [20,21]. Engraftment of human peripheral blood lymphocytes (PBL) in mice has been reported using two NOD/scid strains, one with a truncated mutation (NOG) and one with a null mutation (NSG) of the IL-2-receptor γ-chain, using either human CD34^+ ^stem cells or peripheral blood mononuclear cells (PBMC) [22,23]. Our main interest was to assess T cell responses using autologous DC-based vaccines. Humanized mice reconstituted with PBMC were considered most amenable for such studies since they enabled purified monocytes to be used for the generation of autologous mDC. Engraftment of PBMC places constraints on the vaccination protocol due to the development of confounding xenogenic responses over time. These limitations can be bypassed using CD34^+ ^stem cells for NSG reconstitution [24], but the acquisition of CD34^+ ^cells, along with autologous monocytes for DC generation, is more difficult.

To assess our model, we used NOD/scid IL2Rg^null ^(NSG) mice to engraft human PBMC and performed vaccination experiments using 3- and 7-day mDC prepared *in vitro *from the same donors. Additionally, we compared cocktail 4C with our maturation cocktail containing poly (I:C) as a TLR3 agonist and R848 as a TLR7/8 agonist (5C+R848). Our comparisons showed that variably matured DC had different impacts on induction of antigen-specific CD8^+ ^T cells *in vivo*, opening the door to use this humanized mouse model to assess variations in DC vaccines *in vivo*.

## Methods

### Preparation of PBMC for engraftment of NSG mice and *in vitro *generation of mDC

Peripheral blood of healthy donors was prepared as described [18]. In brief, fresh whole blood from healthy adult donors collected with preservative free heparin was diluted (1:3) with low endotoxin PBS (PBSle) (Biochrom) and the leukocyte fraction was enriched using standard ficoll gradient centrifugation. The interface was harvested and washed twice with PBSle. Afterwards PBMC were used directly for engraftment using intravenous injection of cells in PBSle or as sources of mDC [5,18]. For DC preparation, monocytes were isolated via flask adhesion and cells were cultured for 2 or 6 days with IL-4 and GM-CSF. Afterwards, maturation cocktails were added as described [18] using 4C and 5C+R848 cocktails. See Table 1 for cytokine concentrations. DC were harvested after 24 h, loaded with antigen and incubated for an additional 6 h before injection into mice reconstituted with human PBMC. Collection of blood from healthy donors was approved by the "Ethics Board of the Medical Faculty of the Ludwig-Maximilians-University" Munich, Germany and donors gave informed consent.

**Table 1:** Composition of cocktails used for DC maturation

Cocktail	Inflammatory cytokines/interferons	Other additives	TLR-ligands
4C	TNF-α, IL-1β, IL-6	PGE_2_	
5C + R848	TNF-α, IL-1β, IFN-γ	PGE_2_	poly(I:C), R848

The following concentrations were used in the maturation cocktails:

4 C: TNF-α (10 ng/ml); IL-1β (10 ng/ml); PGE_2 _(1000 ng/ml); IL-6 (15 ng/ml)

5 C + R848: TNF-α (10 ng/ml); IL-1β (10 ng/ml); PGE_2 _(250 ng/ml); IFN-γ (5000 U/ml); poly (I:C) (20 ng/ml); R848 (1 μg/ml)

### Surface phenotyping of mDC

Labeling of harvested mDC was performed with monoclonal antibodies against CD14 (FITC, clone MΦP9), CD80 (PE, clone L307.4), CD274 (B7-H1, FITC, clone MIH1) (all BD Biosciences) and CD83 (PE, clone HB15a) (Immunotech). After a 60 min incubation step, cells were washed and analyzed using a FACSCalibur instrument (BD Biosciences). Post-acquisition data was analyzed using the FlowJo 8 Software (Tree Star, Ashland, OR).

### Signal 3 assay of cytokine secretion

2 × 10^4 ^harvested and washed mDC were cocultured with 5 × 10^4 ^CD40L-expressing murine fibroblasts as a mimic for interaction with activated T cells, as described previously [18]. Following 24 h-coculture, supernatants were collected for detection of IL-10 and IL-12(p70).

### Cytokine secretion measurement by ELISA

Collected supernatants of signal-3-assay cocultures were analyzed for the amounts of secreted IL-10 and IL-12(p70) using standard ELISA. The amount of human IFN-γ was also measured in serum samples of immunized mice using standard ELISA kits (BD Systems) according to manufacturer's instructions. Colorimetric substrate reaction with tetramethylbenzidine and H_2_O_2 _was stopped with H_3_PO_4 _and measured at 450 nm and wavelength correction at 620 nm and analyzed with "easy fit" software (SLT).

### Engraftment PBMC in NSG mice

For the 9-week reconstitution protocol, mice were irradiated with a sub-lethal dose of 100cGy one day before intravenous injection of 1 × 10^6 ^human PBMC, while the 4-week protocol used a single intravenous injection of 10 × 10^6 ^PBMC, without irradiation. Mice were vaccinated first on day 42 or day 14 after reconstitution, respectively. The Bavarian State authorities approved all animal experiments.

### Electroporation of mDC and vaccination of humanized mice

Specific antigen was introduced to mDC in the form of *in vitro-transcribed*-RNA (*ivt*-RNA) encoding full length protein, as described [5]. Electroporation of mDC was performed using 48 μg of *ivt*-RNA, prepared from linearized T7-promotor-containing plasmid, with the mMESSAGE mMACHINE T7 kit (Ambion), following the manufacturer's instructions. We used the plasmid pcDNAI containing MART-1 cDNA. Electroporation was performed as described in [5] and mDC were cultured 6 h following electroporation. Afterwards, mDC were injected immediately or cryopreserved for later vaccination. DC (1 × 10^6^) were given twice intravenously, with a one-week interval between injections. After 7-14 days, mice were sacrificed and splenic populations containing human lymphocytes prepared for *in vitro *characterization of immune responses.

### Functional analysis and flow cytometry

Splenic populations with human cells were also cultured in RPMI 1640 medium supplemented with 10% fetal calf serum, 2 mM L-glutamine, 1 mM sodium pyruvate, 1 mM non-essential amino acids and 5 μM beta mercaptho-ethanol, over a 7-day period, in the presence of 50 IU/ml human IL-2 and 0.1 μg/ml OKT-3, prior to chromium-release assays. Cytotoxic assays were performed as described [18]. Tumor cell lines used as targets were cultured as described [18,25]. For flow cytometry, splenic populations were cultured overnight without supplementations of IL-2 and OKT-3 and stained using an HLA-A2/MART-1_25-36_-specific multimer (peptide sequence: ELAGIGILT; PE-conjugated; kindly provided by D. Busch, Technical University of Munich) or with an HLA-A2/CMVpp65-specific control multimer for 20 minutes, followed by washing and staining with specific CD3 (PerCP; clone SK7, BD) and CD8 (APC; clone SK1, BD) or with directly labeled CD3, CD4 (PE; clone 13B8.2, Immunotech), CD8, CD62L (FITC; clone SK11, BD) antibodies. Measurement was performed using a LSRII machine and FlowJo software for analyses. Staining of blood samples was performed accordingly, including an erythrocyte lysis step prior to staining with antibodies.

## Results

### Human PBMC rapidly engraft in NSG mice

**Figure 1:**
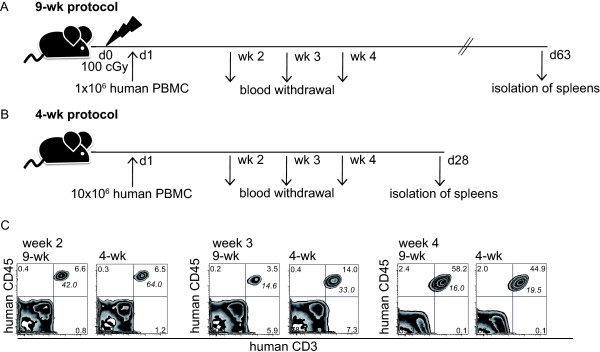
**Efficacy of engraftment of human PBMC in NSG mice**. (**A**) Schema of 9-wk engraftment protocol. Mice were irradiated 24 h prior to i.v. injection of 1 × 10^6 ^human PBMC and spleens were isolated on day 63. (**B**) Schema of 4-wk engraftment protocol starting with i.v. injection of 10 × 10^6 ^human PBMC on day 1. Isolation of spleens was performed on day 28. (**C**) Representative staining of CD3 vs. CD45 in blood withdrawals of individual mice engrafted with the 9-wk or the 4-wk protocol on week 2, 3 and 4. Italic numbers given beside the CD3/CD45-positive population indicate the percentage of CD62L-positive cells within the double-positive fraction.

To develop a humanized mouse model to study DC vaccination *in vivo*, we used NSG mice xenografted with human PBMC. Initially we compared two published engraftment methods, with minor modifications [20,26]. As shown in Figure 1A and 1B, the protocols differed in the length of 9 weeks (9-wk) (Figure 1A) or 4 weeks (4-wk) (Figure 1B). For the 9-wk protocol, mice were irradiated with a sub-lethal dose of 100 cGy, 24 h prior to injection of 1 × 10^6 ^human PBMC. By contrast, the 4-wk protocol utilized non-irradiated recipients which received 10 × 10^6 ^PBMC.

Weekly analysis of CD3/CD45-positive cells in blood withdrawals of non-vaccinated mice showed T cell engraftment by both protocols (Figure 1C). At week three, faster engraftment was seen using the 4-wk protocol. This was accompanied by a higher percentage of CD62L-positive cells in the CD3/CD45-positive fraction, indicated as italic numbers in the FACS plots. Both protocols showed comparable engraftment at week four.

### DC vaccines differ in phenotype and IL-12 secretion

**Figure 2:**
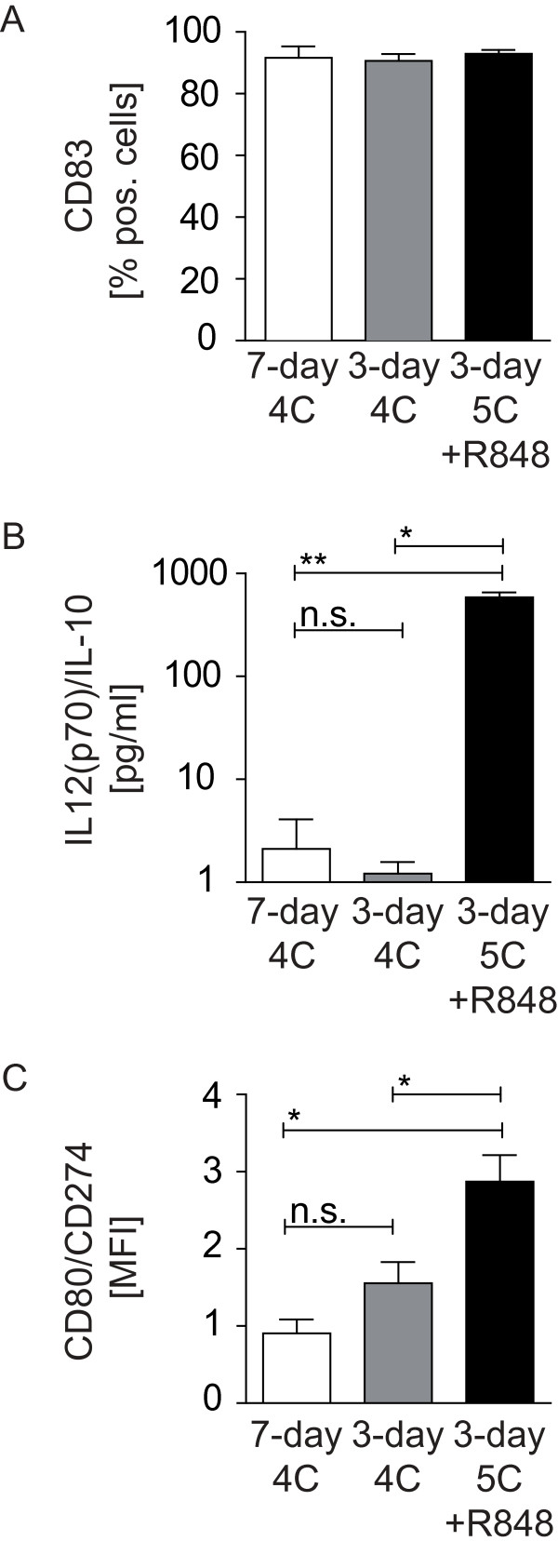
**Phenotypic and functional analysis of differently matured DC**. (**A**) Percent of CD83-positive cells measured on 7-day, 4C-matured DC (n = 6) as well as 3-day, 4C- and 5C+R848-matured DC (n = 4). Percent of CD14-positive cells ranged between 0.9 and 5.7 percent in the tested populations. (**B**) Ratio of secreted IL-12(p70) to IL-10 assessed by standard ELISA of signal-3-assay supernatants. Analyzed DC were matured using the 7-day, 4C protocol (n = 7) or the 3-day protocol in combination with 4C or 5C+R848 mediated maturation (n = 4). (**C**) Ratio of the mean fluorescence intensity of CD80 to CD274 (B7-H1) assessed on 7-day, 4 C generated mDC (n = 3) as well as 3-day DC matured with 4C or 5C+R848 cocktails (n = 6). Significance was ascertained using a two-tailed Mann-Whitney *U *test and *p*-values were defined as following: ** = *p *< 0.005; * = *p *< 0.05; n.s. = *p *> 0.05.

We have previously reported on the development of monocyte-derived DC-based vaccines that use mature cells produced in a shorter 3-day period instead of the more standard 7-day culture duration [5]. In addition, we also reported on the implementation of TLR3 and TLR7/8 activation signals using synthetic agonists [17,18]. Both modifications enhanced the capacity of mDC to induce antigen-specific T cell responses *in vitro *[5,17,18]. These variations were selected for assessment in reconstituted mice. To monitor the *in vitro *maturation process of DC for later administration to humanized mice *in vivo*, we determined expression of the markers CD14 and CD83 on human DC generated via 3-day or 7-day culture periods. Furthermore, 3-day DC were compared after maturation with two different cocktails (4 C or 5 C + R848). As reported previously, all three mDC variants showed increased expression of CD40, CD80, CD86, HLA-DR and CCR7 to varying degrees (data not shown) [5,17,18]. The maturation status of the three mDC variations was compared by determining percentages of CD83-positive cells. Significant differences were not detected among the three variants (Figure 2A). As a second parameter, we assessed the amounts of IL-10 and IL-12(p70) secreted after T cell encounter, mimicked in a signal-3-assay using CD40L-expressing murine fibroblasts for DC stimulation. The amounts of IL-12(p70) to IL-10 were compared by calculating the ratio for each DC phenotype. These confirmed published results that DC matured with 4C failed to secret IL-12(p70). In contrast, the ratio of IL-12(p70) to IL10 was significantly increased for 5C+R848-matured, 3-day DC due to enhanced secretion of IL-12(p70) [17,18] (Figure 2B). As a third feature, the mDC were analyzed for their expression profile of the positive costimulatory molecule CD80 compared to that of the inhibitory costimulatory molecule CD274 (B7-H1). As previously published, we observed changes in the ratio of CD80 to CD274 between DC cultured for 3 days and 7 days, due to increased expression of CD274 over time [5]. Furthermore, we detected an additional increase in the expression levels of CD80 when DC were matured with 5C+R848 [18] (Figure 2C).

Taken together these results indicated that 5C+R848-matured, 3-day mDC displayed an optimal phenotype for the induction of antitumor immunity based on assessments *in vitro*. Our functional studies *in vitro *also supported this contention [5,17,18]

### DC vaccination does not impact on engraftment

**Figure 3:**
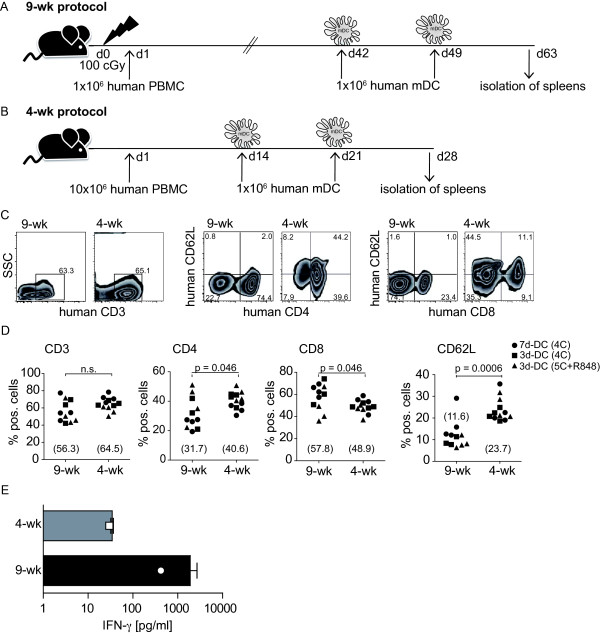
**Influence of vaccination on the engraftment efficacy of human PBMC**. (**A**) Schema of 9-wk engraftment protocol in which vaccination was performed using 1 × 10^6 ^mDC given on day 42 and day 49. Spleens were isolated on day 63. (**B**) Schema of 4-wk engraftment protocol using injection of 1 × 10^6 ^mDC on day 14 and day 21. Spleens were isolated on day 28. (**C**) Representative examples for CD3 vs. SSC, CD4 vs. CD62L and CD8 vs. CD62L (from left to right) of flow cytometry analysis comparing engraftment efficacy of 9-wk (left of each block) and 4-wk (right of each block) schemes. (**D**) Statistical analysis of engraftment efficacy comparing 9-wk and 4-wk protocols and mice vaccinated using 7-day 4C-matured DC (circles), 3-day 4C-matured (squares) or 3-day 5C+R848-matured (triangles) DC; means are given in brackets. Statistical analyses were performed using a two-tailed Mann-Whitney test and *p *< 0.05 were considered significant. Engraftment experiments were performed at least 3 times with similar results. (**E**) Amounts of human IFN-γ in sera of vaccinated mice engrafted with the 9-wk or 4-wk protocol, respectively, were assessed using a standard ELISA. Depicted are means and SEM of 8 mice generated in two individual experiments. The open square and open circle represent levels in individual non-vaccinated mice.

Because the engraftment protocols had substantial differences with respect to time, numbers of PBMC and irradiation of NSG recipients, it was important to determine whether the engraftment characteristics of mice were altered by vaccination. Further, we compared the DC variants since different DC phenotypes might impact directly on T cell engraftment. Mice engrafted with the 9-wk protocol were first immunized with the DC vaccines 6 weeks later (Figure 3A) while the first immunization was performed two weeks following engraftment in the 4-wk protocol (Figure 3B). Vaccination of mice in each group consisted of two intravenous injections of 1 × 10^6 ^mDC, separated by a one-week interval. Freshly prepared mDC from autologous donors were used for the first vaccination and cryopreserved mDC were used from the same production lot for the second vaccination. The characteristics described for the mDC (Figure 2) were not altered by cryopreservation (data not shown) [5].

FACS analysis of splenic cells of vaccinated mice showed comparable engraftment of human CD3^+ ^T cells, as illustrated for representative animals of each protocol at the day of harvest (day 63 and day 28), respectively (Figure 3C). The most prominent distinction noted in the mice was the higher numbers of CD4^+ ^and CD8^+ ^cells expressing CD62L, which is characteristic of naïve lymphocytes, in the mice reconstituted by the 4-wk protocol (Figure 3C). Mean percentages of CD3^+ ^T cells were 56.3% and 64.5% for the 9-wk and 4-wk groups, respectively (Figure 3D). As a group, mice reconstituted with the 9-wk protocol had higher mean percentages of CD8^+ ^T cells (57.8% vs. 48.9%), whereas mice reconstituted with the 4-wk protocol showed greater numbers of CD4^+ ^T cells (31.7% vs. 40.6%), reflecting more the normal situation in humans. The strong distinction noted in Figure 3C with respect to CD62L expression was consistently reflected in the two groups of mice (11.6% vs. 23.7%) (Figure 3D). This indicates that at the time of analysis the majority of lymphocytes of the 9-wk group had been activated *in vivo*, whereas a fraction of naïve cells remained in the 4-wk group. It is important to note that differences were not seen with the use of different vaccine variants, but rather reflected differences in the engraftment protocol as a whole.

Furthermore, we observed signs of graft-versus-host disease (GvHD) approximately 5 weeks after engraftment using the 9-wk protocol (not shown), which resulted in untimely death of several mice. Mice showed a strong loss of weight, fur loss and thin red skin, characteristics previously described for a xenograft GvHD model [27]. Additional analysis of the sera of sacrificed mice demonstrated increased levels of IFN-γ in mice generated with the 9-wk protocol (range: 500-5000 pg/ml) compared to sera of mice generated with the 4-wk protocol (< 50 pg/ml) (Figure 3E). Based on these observations, we selected the 4-wk procedure for further DC vaccination studies. Since the engraftment characteristics of vaccinated mice were comparable to non-vaccinated animals, it can be concluded that the DC as applied did not impact on engraftment.

### DC variants induce different levels of MART-1-specific immune response

**Figure 4:**
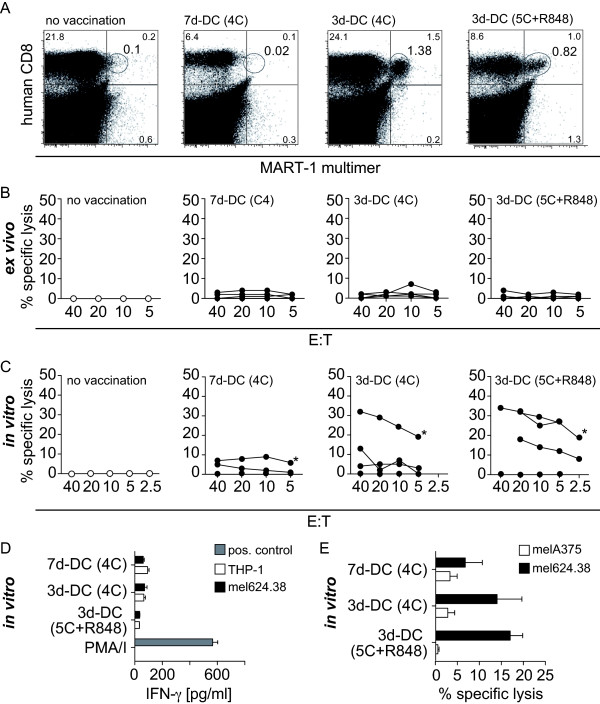
***In vivo *priming using DC-based vaccination**. (**A**) MART-1-specific multimer staining versus CD8 staining of *in vivo*-primed human T cells. Differently matured DC of an HLA-A2^+ ^donor, electroporated with MART-1 *ivt-*RNA, were used for vaccination. Staining was performed one day after spleen isolation. (**B**) Killing capacity (% specific lysis) of human lymphocyte populations, shown in A, tested individually in a chromium-release assay 24 h after isolation (*ex vivo*). 2 × 10^3 ^mel624.38 target cells were incubated with varying numbers of effector cells. Specific lysis of non-immunized mice is shown as open circles while filled circles represent specific lysis of lymphocytes from immunized mice. THP-1 cells (HLA-A2^+^, MART-1^-^) cells were not recognized (data not shown). (**C**) Killing capacity (% specific lysis) human lymphocyte populations cultured *in vitro *after isolation from individual mice and tested separately in a chromium-release assay at day 7 after isolation. Responses of non-immunized mice are shown as open circles while filled circles represent responses of immunized mice. Individual mice shown in (**A**) are indicated with * and the analyzed lymphocyte populations correspond to the populations tested in B. THP-1 (HLA-A2^+^, MART-1^-^) and K562 (HLA-A2^-^, MART-1^-^) cells were not recognized (data not shown). (**D**) Amount of secreted IFN-γ by human lymphocyte populations after stimulation with mel624.38 cells following *in vitro *culture for 7 days. Analysed cell populations correspond to those tested in B and C and were analysed on the same day as C. Values are given as means of four mice with SEM and PMA/I stimulation served as the positive control. (**E**) Specific lysis of melA375 (HLA-A2^+^, MART-1^-^) and mel624.38 (HLA-A2^+^, MART-1^+^) melanoma cell lines as target cells. Shown are means (SEM) of four mice vaccinated with MART-1-expressing DC populations using the 4-wk protocol.

To assess if antigen-specific T cell responses could be detected in mice reconstituted and vaccinated with the 4-wk protocol, mDC were used for vaccination that expressed the complete protein for the melanoma-associated antigen MART-1. These mDC were prepared from monocytes of an HLA-A2^+ ^donor that were electroporated with *ivt*-RNA encoding full length MART-1 protein. NSG mice reconstituted with autologous HLA-A2^+ ^PBMC were vaccinated twice as depicted in Figure 3B and seven days later splenic populations were analysed by FACS using a MART-1-specific multimer to enumerate CD8^+ ^multimer-binding cells within the human CD3^+ ^T cell fractions. 7-day cultured mDC usually failed to induce multimer-positive cells above the background levels of non-vaccinated animals, while 3-day mDC derived using either 4 C or 5 C + R848 induced multimer-positive cells to varying degrees in individual mice (Table 2). Examples of multimer-binding to CD8^+ ^human lymphocytes present in isolated splenic populations are shown in Figure 4A. Direct analysis of these splenocyte populations *ex vivo *failed to demonstrate detectable cytotoxic activity directed against HLA-A2^+ ^MART-1^+ ^melanoma tumor cells as targets in a standard chromium release assay (Figure 4B). It was also not possible to measure secretion of interferon-gamma (IFN-γ) after coculture for 24 hours with these melanoma cells (Figure 4D).

**Table 2:** Multimer-positive cells after vaccination with MART-1-pulsed mature DC

mDC phenotype	7d-DC (4C)	3d-DC (4C)	3d-DC (5C+R848)
	0.02	0.38	0.80
	0.28	1.38	0.82
	0.00	0.18	0.32
		0.08	0.15

mean	0.10	0.51	0.52
SEM	± 0.09	± 0.30	± 0.17

Therefore, splenocytes from the vaccinated mice were cultured for one-week *in vitro *in the presence of human anti-CD3 antibody and human IL-2 to enrich the human T cells before again assaying their cytotoxic function. Enrichment efficacy was controlled on day 6 of *ex vivo *culture and resulted in a purity of at least 80% human CD3^+ ^T cells (data not shown). The subsequent chromium-release assay assessed specific responses using mel624.38 cells (HLA-A2^+^, MART-1^+^) as positive target cells and THP-1 cells (HLA-A2^+^, MART-1^-^) and melA375 cells (HLA-A2^+^, MART-1^-^) as negative controls. As an additional control, K562 cells were used to determine potential cytotoxic activity of human NK cells. We did not observe specific lysis of any of these three MART-1^- ^target cells in any of the tested samples (data not shown). As indicated by the multimer-staining results, human lymphocytes from mice immunized with 7-dayDC showed no clear lysis of tumor cells *in vitro *(Figure 4C, *indicates mice depicted in Figure 4A). In mice immunized with 3-day DC matured by 4C, human lymphocytes of only one mouse of four in this experiment showed strong specific lysis of the MART-1^+^-cell line, although splenic populations of three of the mice were positive for multimer staining (Table 2). In contrast, 3-day DC matured with 5C+R848 induced MART-1-specific responses in three of four mice. The specificity of mel624.38 (HLA-A2^+^, MART-1^+^) killing was verified by demonstrating no recognition of melA375 (HLA-A2^+^, MART-1^-^) melanoma cells (Figure 4E), nor were THP-1 and K562 cells recognized as targets (data not shown). The failure to measure any killing with human CD3^+ ^T cells cultured from spleens of non-vaccinated animals served to demonstrate that the positive responses detected in the two groups that were vaccinated with 3d-DC were not due to non-specific activation of MART-1-specific T cells through *in vitro *culture, despite the absence of specific MART-1 antigen.

**Figure 5:**
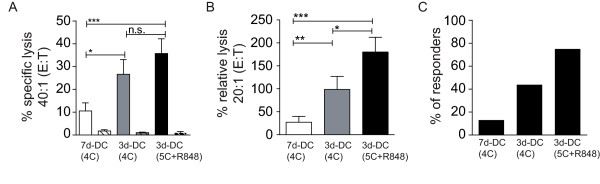
***In vivo *priming efficiency of MART-1-positive DC**. (**A**) Specific lysis of mel624.38 (HLA-A2^+^, MART-1^+^) and THP-1 (HLA-A2^+^, MART-1^-^) target cells by splenic-derived human lymphocytes isolated from mice vaccinated with various mDC types. Depicted are means of results from 16 mice total per group generated in 4 individual experiments and tested for significance using a two-tailed Mann-Whitney test (p values were considered as following * *p *< 0.05; ** *p *< 0.005; *** *p *< 0.0001) (**B**) Relative lysis of results shown in (**A**) adjusted for numbers of human CD8^+ ^T cells. To account for individual experiments and mice, values were adjusted at an E:T of 20:1 as follows:% rel. specific lysis = % spec. lysis/(% CD8/100). Given are means with SEM (* *p *< 0.05; ** *p *< 0.005; *** *p *< 0.0001, assessed by a two-tailed Mann-Whitney test). (**C**) Percentage of responding mice after vaccination with MART-1-expressing mDC of an HLA-A2^+ ^donor. Mice were considered as responders when human lymphocytes, isolated and cultured from spleens, showed a specific lysis of HLA-A2^+^, MART-1^+ ^target cells (mel624.38) higher than 20% at an E:T of 40:1. Analyzed were 4 individual experiments with a total of 16 mice in each group.

Collective results of chromium-release assays, performed with cells cultured for 7 days after spleen isolation, were evaluated for four independent reconstitution experiments using the different MART-1-protein-expressing mDC variants for vaccination of mice engrafted with PBMC derived from HLA-A2^+ ^donors. Specific cytotoxicity of the T cell cultures derived from splenic populations of the three vaccination groups are illustrated at an E:T of 40:1, analyzing the specific lysis of mel624.38 cells as positive targets and THP-1 cells as negative controls (Figure 5A). The highest specific lysis was consistently observed with T cells from mice vaccinated with 3-day, 5C+R848 mDC, followed by 3-day, 4C mDC. Little or no specific lysis was seen using cells from mice vaccinated with 7-day, 4C DC, corresponding with the negative results of multimer staining.

To account for variations in actual numbers of human CD8^+ ^T cells among the lymphocytes cultured *ex vivo*, we calculated a percentage relative lysis normalized to the percentage of CD8^+ ^T cells in the individual cultured splenic populations. Mean relative lysis at an E:T of 20:1 for a total of 16 mice per group is given in Figure 5B. These results demonstrated that 3-day DC matured with 5C+R848 were superior in their capacity to induce human antigen-specific CD8^+ ^CTL.

We designated mice as responders if the *in vitro *cytotoxicity test showed a minimal specific lysis of 20% at an E:T of 40:1. For mice vaccinated with 3-day, 5C+R848-matured DC, we observed 75% responders (12 of 16 analyzed mice) (Figure 5C). Vaccination using 3-day, 4C-matured DC resulted in 44% responders (7 of 16 mice), whereas 7-day, 4C-matured DC resulted in only 13% responders (2 of 16 mice).

## Discussion

While humanized mouse models have been efficiently used to evaluate pathogen-directed vaccination strategies, as nicely reviewed by Münz and colleagues [28], few studies describe the use of humanized mice to assess the efficacy of human DC vaccines [20,21]. The ability to carry out *in vivo *comparisons of new DC vaccine approaches would be of substantial interest for design of clinical trials.

We have developed a mouse model that is simple and rapid based on the NSG mouse strain to investigate various human DC-based cellular therapeutics. By using NSG mice that were engrafted with human PBMC within two weeks, we successfully avoided the GvHD reaction that was observed using a 9-wk engraftment protocol, which became apparent in most mice as early as week five post PBMC injection. Although engraftment of human cord blood-derived stem cells would result in reduced GvHD, the feasibility of obtaining stem cells and DC from the same donor is very limited. In addition, testing patient-derived DC with lymphocytes of healthy HLA-matched donors is also difficult due to limitations in finding suitable HLA matches. By adapting a 4-wk protocol using 10 × 10^6 ^PBMC for engraftment, followed by two vaccinations with 1 × 10^6 ^mDC, we were able to induce and detect allo-reactive (data not shown) as well as autologous antigen-specific responses in donor T cells from humanized NSG mice.

To test this NSG model, we compared three DC vaccine formulations that were previously characterized extensively *in vitro *using MART-1 as an antigen. We selected MART-1 for evaluation of antigen-specific responses because of the known high frequency of CD8^+ ^T cells responding to this antigen in most healthy individuals, which can be detected easily with MHC-multimers. In these studies, we did not analyze whether MART-1-specific CD4^+ ^T cells were also present, but the good reconstitution of human CD4^+ ^T cells in the NSG mice generated using the 4-wk protocol may indeed allow their induction through appropriate vaccination.

The use of MART-1 as a surrogate antigen, like lymphocytic choriomeningitis virus (LCMV) and ovalbumin (OVA) in many murine models, is suitable for many comparisons. Further investigations will be required to determine if responses to additional antigens can be detected *in vivo*. Here it may be necessary to reconstitute mice with PBMC of cancer patients to access higher frequency memory responses to some TAA.

The DC populations that we compared *in vivo *varied in time of generation (3d vs. 7d) and signals for maturation (plus/minus TLR3, TLR7/8). In the humanized mice, 7-day DC failed to induce potent immune responses, while mDC generated in a 3-day period using the 4C cocktail induced good responses in 44% of mice. Furthermore, DC matured with the TLR3 and TLR7/8 agonists not only gave enhanced immune responses in individual mice, but also increased the numbers of positive responders within the cohort to 75%. It is not known whether failure to induce immune responses in 25% of mice is due to deficiencies in vaccination or reflects variations among mice, for example, in qualitative or quantitative levels of reconstitution. Nevertheless, the three vaccine groups as a whole allowed distinctions to be drawn with respect to the three vaccine variations analyzed here, which reflected the hierarchy we previously found in these mDC variations *in vitro *[5,17,18]. It was interesting to note that multimer-positive cells were often detected in splenic populations after vaccination with 3-day 4C-matured DC, but parallel killing capacity was not always detected in all *in vitro *cultures of human CD3^+ ^T cells. Further studies of human cells isolated directly from splenic populations may shed light on this discrepancy. For example, it remains to be determined whether this represents anergy in some antigen-specific T cells or perhaps an increased prevalence of regulatory cells that suppress immune reactivity *in vitro*.

In the experiments reported here we used only intravenous injection of DC. Further studies will be required to determine if subcutaneous or intradermal injection will allow induction of immune responses. DC migration to lymph nodes may not function well in NSG mice due to the poor structure of their lymph nodes. The migratory capacity of human mDC in a xenogenic tissue microenvironment also remains an open question that will strongly impact on immune responses. Therefore, it is fortunate that intravenous injection of mDC allowed immune responses to be detected in human lymphocytes residing in the spleens of reconstituted mice. Studies regarding frequency of DC application and time span of vaccination will be limited by the appearance of xenoreactive cells, which were prominent already after five weeks in mice reconstituted by the 9-wk protocol.

## Conclusions

In conclusion, the NSG humanized mouse model system described here enables investigation of therapeutic cell reagents in an *in vivo *setting. This model easily allows the assessment of the potential of various human DC preparations to activate autologous antigen-specific T cells in an *in vivo *setting if one uses PBMC from HLA-A2^+ ^donors and MART-1 as a surrogate antigen. The relatively small numbers of cells required for engraftment and vaccination should allow some studies using patient material. In particular, this model should enable comparisons among different DC vaccine types to be rapidly assessed *in vivo*. Because the human CD3^+ ^T cells can be recovered from the spleens of mice and cultured *ex vivo*, further studies are applicable. For example these studies could assess lymphocyte subsets, Th1/Th2 polarization, as well as presence of regulatory populations and the impact of DC vaccination on their functions.

## Abbreviations

4C: Four-component cocktail; 5C+R848: Five-component cocktail plus R848; *ivt*-RNA: *In vitro-transcribed*-RNA; GvHD: Graft versus Host Disease; NSG: NOD/scid IL2Rg^null^; TLR: Toll-like receptor.

## Competing interests

The authors declare that they have no competing interests.

## Authors' contributions

SS designed and performed the experiments and drafted the manuscript. DJS and BF designed the experimental concept and prepared the final manuscript. All authors read and approved the final manuscript.
